# Lithophagia as a clue for celiac disease: a case report and literature review

**Published:** 2017

**Authors:** Mosayeb Shahryar, Iraj Shahramian, Seyed Mohsen Dehghani, NoorMohammad Noori, Maryam Ataollahi

**Affiliations:** 1 *Children and Adolescents Health Research Center, Zahedan Medical University, Zahedan, Iran*; 2 *Department of Pediatrics, Zabol University of Medical Sciences, Zabol, Iran*; 3 *Gastroenterohepatology Research Center, Shiraz University of Medical Sciences, Shiraz, Iran*

**Keywords:** Lithophagia, Pica, Celiac disease, Iron deficiency anemia

## Abstract

Lithophagia is a type of pica that might be resulted from Iron Deficiency Anemia (IDA) which is the frequent presenting signs of Celiac Disease (CD). A 5-year-old child with a two year history of the lithophagia with a, refractory IDA, abdominal distention and constipation. The child did not grow well and had failure to thrive. With suspicion to CD, TTg IgA level was measured and due to an incearse of TTg IgA level the patients were undergone esophagogastrodeudonoscpy and jejunal biopsy. The biopsy showed severe villous atrophy and an increase in limphoplasma cells. Biopsy confirmed diagnosis of CD and glutten free diet was initiated finally. Six months after diagnosis and commencing the gluten free diet, the lithophagia and constipation in patient eradicated completely. IDA and failure to thrive were improved and the level of TTg IgA was reached to the normal. The case demonstrated the relationship between lithophagia and CD in anemia. Therefore, in the same cases such as our case should be considered CD as the most important causes of lithophagia.

## Introduction

Celiac disease (CD) is an autoimmune mediated chronic inflammatory gluten sensitive bowel disorder with strong evidence of T- cell mediation, occurring in genetically predisposed individuals. (1 ,2). Its prevalence varies in different subgroups from 0.5 to 12 percent (3, 4 ,5).

CD in children could present with extra intestinal signs, such as short stature, delayed puberty, Iron Deficiency Anemia (IDA), Eating Disorders (ED) and Osteopenia (6,7,8,9,10,11). From the extra gastro-intestinal point of view, IDA is the most involvement reported; also this IDA is not responsible for Iron supplementation (8). As mentioned, IDA and eating disorders are signs of malabsorption , even in patients without gastrointestinal symptoms (8,10).

Pica is an irresistible desire for consumption of non-nutritive and unusual substances, such as soil, chalk, gypsum, ice and pebbles or rocks which could be documented in IDA, ED, chronic renal failure, and pregnancy (10,12). Lithophagia , a type of Pica, is an extremely rare reported desire of eating pebbles or stone fragments (12). Some associated complications have been reported with lithophagia, including intestinal obstruction (13) and colon perforation (14).

This report describes a child with long term history of lithophagia with the ultimate diagnosis of CD.

## Case Report

A 5-year-old boy with a two year history of the lithophagia, was treated for IDA several times. He had been diagnosed with hypochromic microcytic anemia, but he showed no response to repeated therapeutic iron supplementation. His history revealed several episodes of abdominal distention and long standing constipation that aggravated with iron supplements. Also, he had temptation to lithophagia. Recently, he developed abdominal pain and distention and no defecation for at least 15 days. On arrival, he seemed chronically ill, but his weight and height were acceptable for his age (height=110Cm, weight=20 Kg); moreover, he belonged to a high socio-economic family.

**Figure 1:**
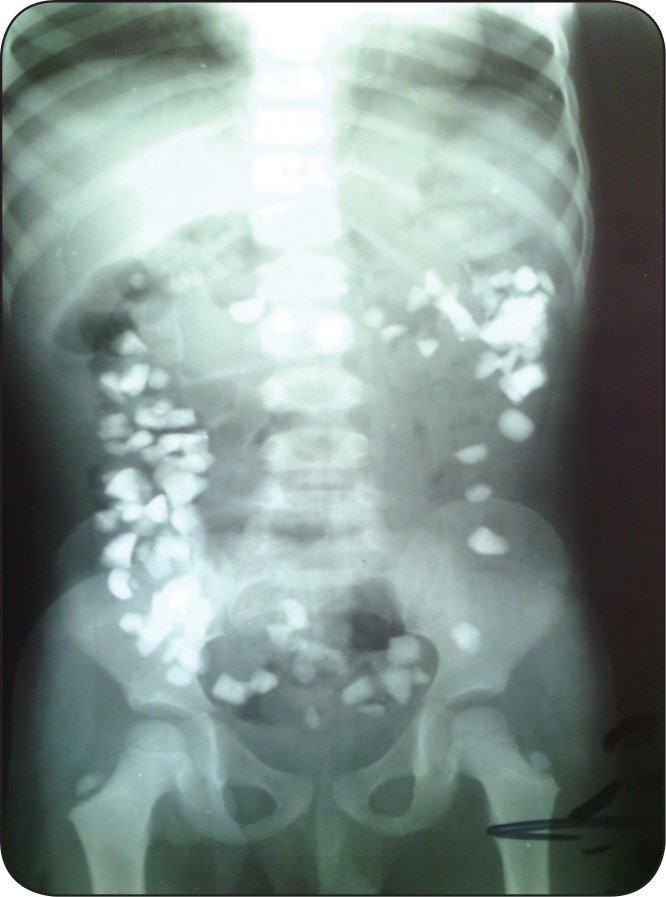
Erect antroposterior radiograph that shows rocks in all parts of colon

**Figure 2:**
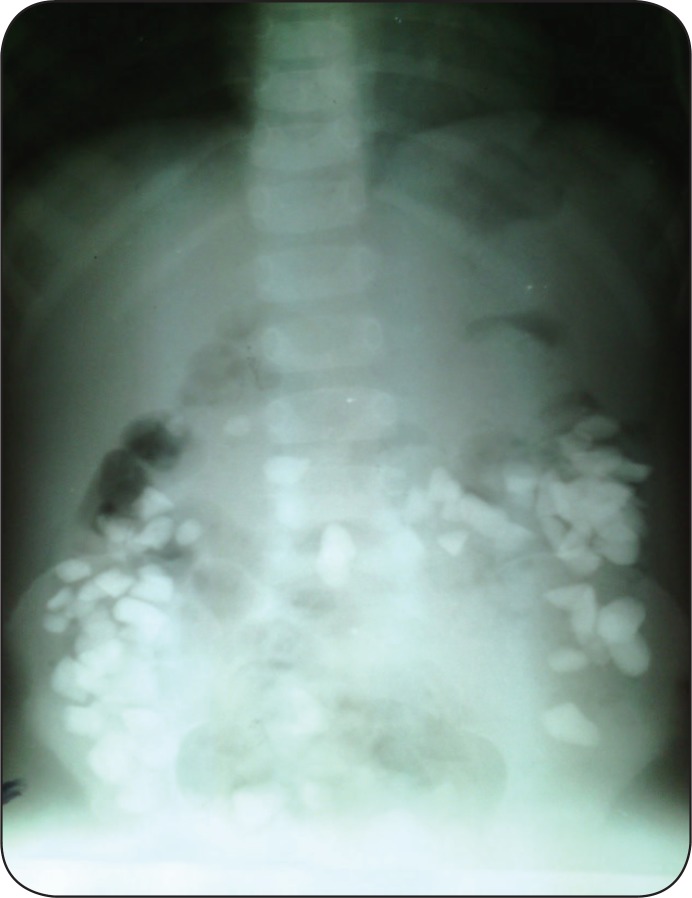
Lay down antroposterior radiograph that shows rocks in all parts of colon

Physical examination documented abdominal distention with decreased bowel sounds but no evidence of organomegaly. Due to his avoidance of rectal examination, plain abdominal X-ray was taken for evidence of fecal impaction, revealing multiple, well-defined radio-opaque foreign bodies in all parts of his colon. (Figures 1, 2)

According to his laboratory assessments, he had heamoglobine 80g/dl, mean circular volume55fl, RBC 3300000, WBC10000, RDW18, total protein 65g/l, and albumin40g/l. With this history of IDA unresponsive to iron supplementations, we were suspicious to CD and total IgA and Anti Tissue Trans-Glutaminase Antibody (TTG-IgA) was measured; TTG-IgA was 400 (normal=20) and total IgA was in a normal range. After that, Esophagogastrointestinal endoscopy was done and multiple biopsy was obtained. Histological examination revealed normal esophagus and stomach but duodenum biopsies documented Marsh-III villus atrophy with an extensive epithelial lymphocyte infiltration in favor of CD.Table(1)

**Figure 3:**
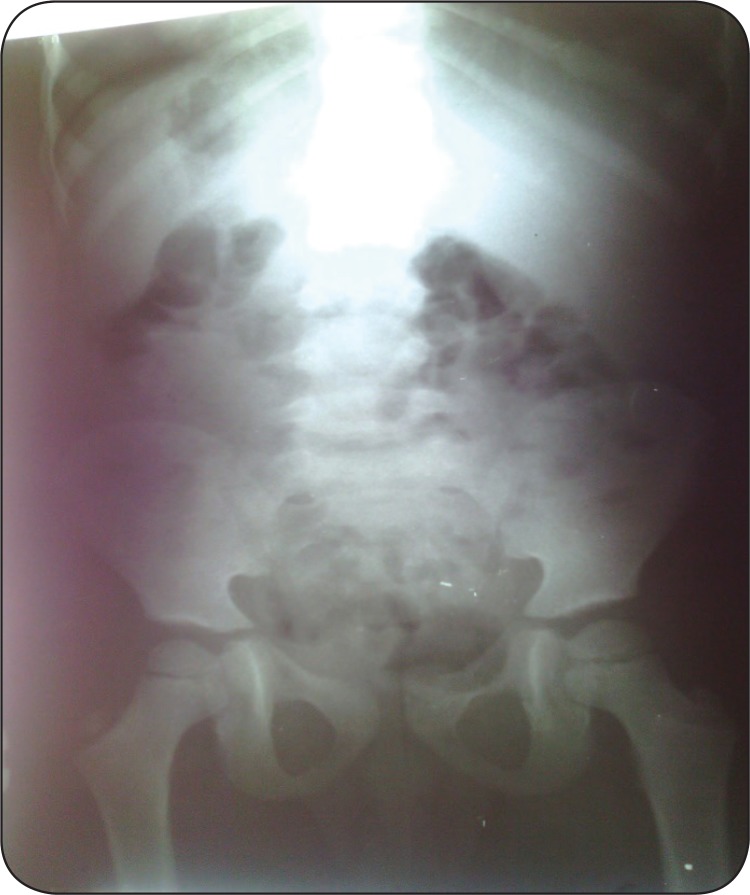
Anteroposterior radiograph after treatment that shows no rocks in colon

The subject commenced lactolose with a dose of 3 ml /Kg/day divided into two equal doses for disimpaction and Gluten free diet. The patient passed rocks without any complication 3 weeks after initiation of lactolose (Figure 3) and his desire to lithophagia was diminished with Gluten free diet.

**Table 1:** Patient’s laboratory data

**Indices**	**Early measure**	**After 6-months**
**Heamoglobin (g/dL)**	80	120
**mean circular volume (fl)**	55	78
**RBC (/µL)**	3300000	4100000
**WBC (/µL)**	10000	7000
**RDW (fl)**	18	14
**PLT (/µL)**	600000	300000
**Albumin (mg/ml)**	4	4.5
**Total protein (mg/ml)**	6.5	7
**TTG IgA (ng/ml)**	400	8
**Total IgA (ng/ml(**	130	125
**Weight (kg)**	20	26
**Length (cm)**	110	112

## Discussion

Celiac disease is a frequently observed genetic disorder with a prevalence of 2.67% in the general population and the characteristic immunologic response to gluten that might be silent. (5,15) There is a documented association between CD and iron deficiency anemia and ED. (9, 10) Pica is an eating disorder presented with consumption of strange non-nutritive substances like soil, chalk, gypsum, ice and pebbles or rocks. (7,9,10)

This case was a 5-year-old patient with acceptable growth and development according to his age that presented with lithophagia, IDA, constipation, and abdominal distention.

Eyad Altamini reported a 4.5 year old girl with lithophagia and IDA that was ultimately diagnosed as CD although that girl had poor weight gain, edema in lower extremities, hypo- albuminemia, chemical and radiological rickets, and growth parameter lower than the 3rd percentile (12). But nowadays there are studies documented CD in patients with normal growth, even obese subjects (7,9).

Stanlely Korman presented a case series of three patients with long standing IDA and lithophagia as Pica that had characteristics of CD in serology and jejunal biopsy; he concluded that CD must be noticed in any child manifesting with Pica and IDA, especially in growth retarded patients (6).

Daniel, et al. demonstrated the relationship between CD and EDs in a series of 10 patients, stating the need for multi-disciplinary evaluation and care for these patients (9).

The clinical picture of IDA varies from case to case and symptoms depend on the severity, underlying disease and patient’s characteristics. A proved symptom of IDA is Pica that is due to reduction of iron – containing enzymes and this pica responds quickly to treatment (8,9). IDA patients without bleeding causes, such as menstruation or occult blood loss, were referred to a gastroenterology consultation; more than half of them had pica that can be an explanation for lithophagia in CD (9). IDA is abundant in digestive pathology with two reasonsof a reduction in quality of life and a consequence of gastrointestinal diseases such as CD. IDA is the most frequent extra-intestinal manifestation of CD, so it is crucial (8). 

Many studies have been reported the relationship between EDs and CD and their association with personal behaviours, other personality disorders and mal-absorption due to CD, as causative factors (9). Consumption of strange substances such as rocks, ice and soil in the domain of EDs has been reported. A scarce report has evaluated the relationship of gastrointestinal pathology in the presentation of EDs and Functional Gastrointestinal disorders. It is greatly documented that Pica could be due to metabololic disturbances but not primary behavioral or psychological disorders (9,10).

Evaluation of this case suggests the necessity of serological and histological evaluation for CD in children with Pica, especially lithophagia along with IDA even if their growth parameters are in a normal range.
